# Review of the impacts of COVID-19 pandemic on the employment of college graduates in China and countermeasures to it

**DOI:** 10.3389/fpubh.2024.1390055

**Published:** 2024-06-07

**Authors:** Huicui Wang, Chun Wang

**Affiliations:** Department of Student Affairs, Qingdao University, Qingdao, China

**Keywords:** COVID-19, college graduate, employment impact, employment promotion measure, China

## Abstract

The employment of college graduates has always been the focus issue of the whole society. Affected by the COVID-19, college graduates are facing a severe employment situation. In the present study, we explore the impacts of the COVID-19 on the employment of college graduates. We explore the employment promotion measures introduced by Chinese government and colleges and universities through a quick review and illustrate the difficulties that college graduates face. Furthermore, the present study explores the impacts of the COVID-19 on five aspects of the employment of college graduates: recruitment demand reduce, employment competition rise, employment channels change, psychological anxiety increase and employment structural contradiction intensify. In addition, we conclude that the employment promotion measures introduced by Chinese government and colleges and universities in respond to the impacts of the COVID-19 on the employment of college graduates have significantly promoted the employment of college graduates to a large extent and we provide implications based on the application of the study. The findings of the present study are of great significance for all countries worldwide to better cope with various similar emergency events, to alleviate employment pressure and to promote better and fuller employment of college graduates.

## 1 Introduction

### 1.1 The impact of the COVID-19 on global economy and employment situation

In 2020, Corona Virus Disease (COVID-19) was considered as a pandemic by the World Health Organization (1). It has caused a severe public health emergency and the whole world has fallen into trouble (2, 3). Affected by the COVID-19 pandemic, the global economy has trapped into difficulty (4, 5). The COVID-19 has resulted in unprecedented disruptions in production, consumption, investment, supply chains, tourism, and trade, and it has brought negative effects on the economy of China and the world and the employment of college graduates (6–9). It has led to massive shutdown and production stoppage of enterprises, which has brought a serious negative impact on social development and economic construction and the whole job market (10). Concerning the impact of COVID-19 on employment rate, labor supply and demand, and market wage level, researchers found that COVID-19 had a dual impact on labor supply and demand. On the one side, affected by the COVID-19, the economic growth rate declined, the demand for labor declined, and the employment rate declined. On the other side, the decline of market wages has influenced the labor participation rate and led to the decrease of the number of employed people (11). Moreover, because of the COVID-19, the recruitment demand, internships and interview opportunities have decreased, leading to a substantial rise in college graduates employment stress (12).

### 1.2 The importance of college graduates’ employment

Since employment is the basis of people’s livelihood, stabling employment represents steadying people’s expectations, livelihood and confidence. College graduates are the major power in the employment market with high human capital, and their employment status has become the key issues for the whole society (13–15). Most importantly, as the employment of college graduates is not only concerned with the significance profits of college graduates themselves and the society, but also concerned with the sustainable development of higher education and the prosperity of the country, therefore, facilitating their better and fuller employment is an vital part of the stable development of the present society and the healthy development of economy (10). The employment of college graduates has become the top priority in the employment field under the epidemic. Both the government and colleges and universities paid close attention to the employment of college graduates and provided them with accurate and effective employment assistance services (16).

With college enrollment expanding, the number of college graduates in China is increasing year by year (17). According to the China Bureau of Statistics, the number of college graduates in 2024 in China reached 11.79 million,^1^ the largest number of graduates in history. As the most dynamic, energetic, and creative group, college graduates play a vital role in our society (10). Chinese government has always attached great importance to the employment of college graduates and has taken a series of measures every year to fully ensure the smooth employment of college graduates. On December 5th 2023, the Ministry of Education issued the notice on doing a good job in the employment and entrepreneurship of 2024 national college graduates,^2^ aiming to improve the employment and entrepreneurship promotion mechanism, promote the quality and efficiency of employment and entrepreneurship, and promote the fuller and higher quality employment of college graduates.

### 1.3 The impact of the COVID-19 on college graduates’ employment situation

Although the COVID-19 is effectively under control, the adverse influence of the COVID-19 on the employment of college graduates will remain for some time (18, 19). In general, the outbreak of the COVID-19, external environment factors, the decline of new jobs, the intensified employment competition, the change of employment channels, the psychological quality of college graduates, the intensified employment structural contradiction, the uncertainty of job hunting, gap between high employment expectation and actual situation lead to the employment situation of college graduates more severe (19). In addition, the COVID-19 has changed college graduates’ job orientation. The security and stability of employment has become the main factor for college graduates to choose a job. Furthermore, the COVID-19 also has a negative impact on the job seeking confidence of college graduates. College graduates are worried about their employment prospects. At the same time, college graduates are eagerly looking forward to the introduction of more employment promotion measures (12). In response to the impact of COVID-19, the Chinese government introduced a slew of employment measures for college graduates to alleviate their employment pressure.

### 1.4 The significance of the study

In the context of the COVID-19, there are several objective issues concerning the employment of college graduates, including delayed graduation, employment difficulties, and settling down problems, which are all extremely worrisome. Rich discussions on epidemic and employment were conducted by many domestic and foreign scholars. Most research concentrates on entire employment status, but few studies focus on key group of college graduates (20). Research on employment promotion measures in emergency events has mainly concerned on how to activate job market measures amid the economic crisis (21–23), and few studies focus on how to activate job market measures in emergency situations caused by the COVID-19. Moreover, the existing studies focus less on the impact of the COVID-19 on the employment of college graduates, and the study on the employment promotion measures of college graduates in the emergency status of the COVID-19 is relatively deficient, which also offers us an opportunity to investigate the employment promotion measures of college graduates under the COVID-19 epidemic and other similar emergency situations.

In the present study, we will investigate the impacts of the COVID-19 on the employment of college graduates and examine the employment promotion measures introduced by Chinese government and colleges and universities, finally make suggestions based on the results of the analysis. Five sections follow this introduction. Section methodology articulates the study approach. Section the impacts on the employment of college graduates caused by the COVID-19 and the employment difficulties faced by college graduates introduces the impacts of the COVID-19 on the employment of college graduates and the difficulties that college graduates face. Section countermeasures taken by Chinese government and colleges and universities in respond to the impacts on the employment of college graduates caused by the COVID-19 introduces employment promotion measures implemented by Chinese government and colleges and universities in respond to the severe employment situation. Section implications provides implications for Chinese government and colleges and universities to improve the employment promotion measures of college graduates. Section conclusion concludes the paper.

## 2 Methodology

To ensure the comprehensiveness and depth of the study, the present study adopted diversified research methods, including literature review method, comparative analysis method, and questionnaire survey method. Through a systematic literature review, the present study reviewed, evaluated and comprehensively analyzed the existing literature, research, and reports at home and abroad related with the impacts of the COVID-19 pandemic on the employment of college graduates. Meanwhile, this study adopted comparative analysis method to compare the employment situation of college graduates before and after the epidemic, elucidating the specific impacts and significant changes of the epidemic on the employment of college graduates.

**Figure 1:**
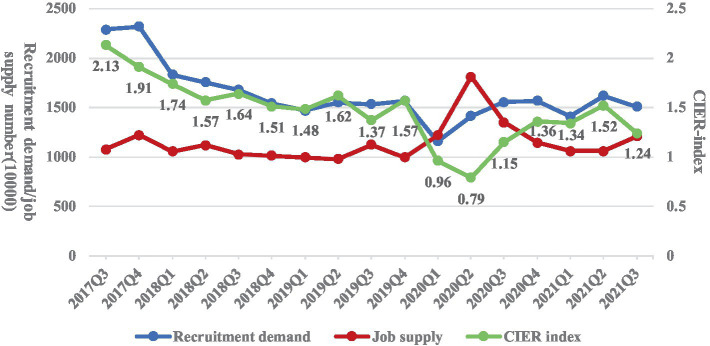
Employment trend of college graduates from 2018 to 2021. Data source: the data is extracted from the Zhaopin recruitment website.

**Figure 2:**
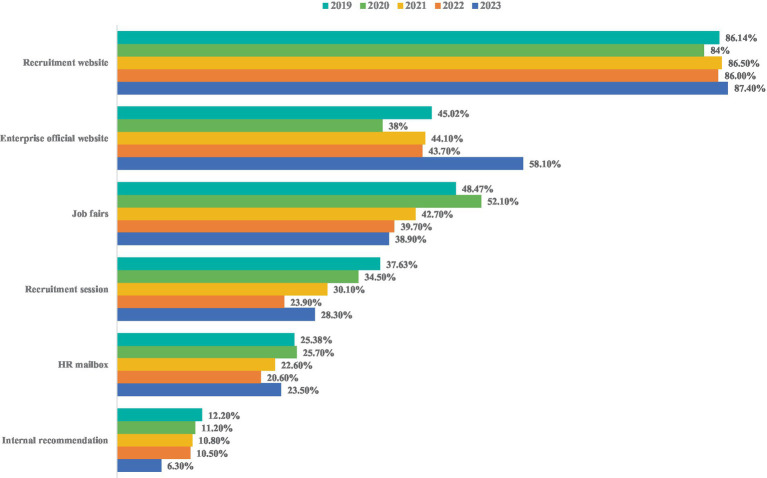
The main channels for college graduates to seek jobs from 2019 to 2023. Data source: the data is extracted from the Zhaopin recruitment website.

**Figure 3:**
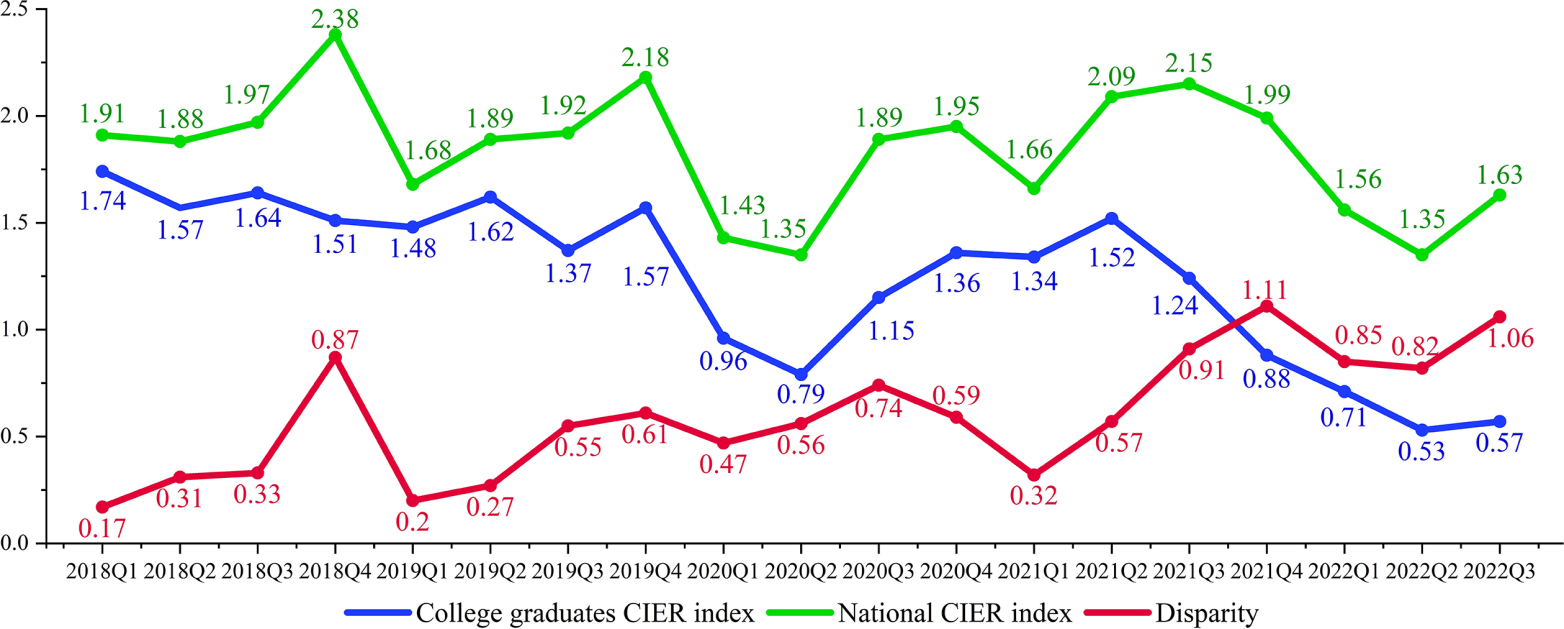
Comparison of CIER index between National and College graduates from 2018 to 2022. Data source: the data is extracted from the Zhaopin recruitment website.

To objectively describe the employment status of college graduates under the influence of COVID-19, this paper uses the big data of Zhaopin recruitment website to analyze the recruitment demand, college graduates’ job supply, employment market prosperity (Figure 1), the main channels for college graduates to seek jobs (Figure 2), and the comparison of CIER index between national and college graduates (Figure 3) (20).

**Figure 4:**
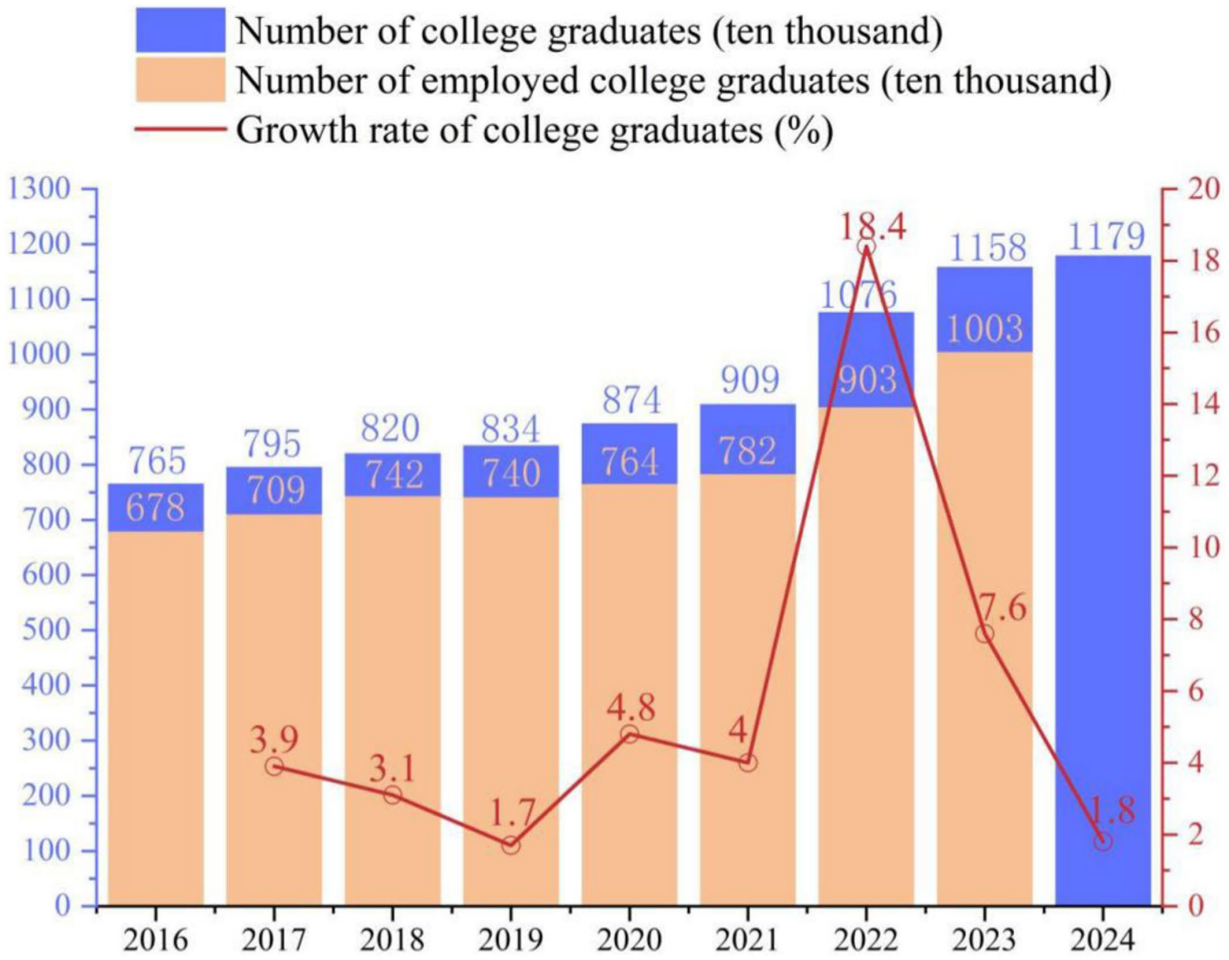
The number of college graduates, number of employed college graduates and growth rate of college graduates in China from 2016 to 2024.

To analyze the specific impacts of the epidemic on the employment of college graduates and elucidate the effectiveness of the employment promotion measures taken by Chinese government and colleges and universities, we analyzed the employment quality of college graduates through questionnaire surveys (Figure 4). The data used in Figure 4 was obtained through the following methods.

This study referenced the “China College Student Survey,” takes higher education institutions recognized by the Ministry of Education in Chinese Mainland as the overall sampling frame and adopted the Analytic Hierarchy Process for correlation analysis. Through orderly hierarchical classification and the construction of analysis matrices, the data analysis is made systematic and scientific. Sampling is conducted in accordance with the principles of multi-stage, hierarchical, and equal probability. The sampling process is divided into three levels: the school level as the first level sampling unit, the subject category as the second sampling unit, and the major as the third sampling unit. In the first stage, schools are sampled based on their level, subject type, and geographical distribution, which are divided into three sampling layers. In the second stage of sampling, the subject category is used as the secondary sampling unit. Among the sampled schools, majors with a number of sufficient samples (sample size greater than or equal to 30 individuals) are randomly selected. In the third stage, majors are the third-level sampling unit, with the total sampling number of each major being no less than 1,000 individuals.

This study conducts a tracking survey of Chinese college graduates from October to December each year, mainly focusing on the employment quality of college graduates from higher education institutions. Undergraduate colleges with a total college graduates population of over 7,000, vocational colleges with a total college graduates population of over 4,000, and industry-specific colleges with a total college graduates population of over 3,000 constitute the main sampling units. The number of surveyed college graduates from 2016 to 2023 was 110,800, 221,000, 256,000, 358,600, 396,300, 416,000, 483,600, and 715,000, respectively. The tracking survey samples in each year are representative, spanning a long period of time, which can accurately reflect the employment situation of college graduates before and after the outbreak of the COVID-19.

## 3 The impacts on the employment of college graduates caused by the COVID-19 and the employment difficulties faced by college graduates

The impacts of the COVID-19 on the employment of college graduates mainly include recruitment demand reduce, employment competition rise, employment channels change, psychological anxiety increase and employment structural contradiction intensify. The introduction of those five impacts is shown below in detail.

### 3.1 Recruitment demand reduce

Since the beginning of the year 2020, the sudden outbreak of COVID-19 not only has dramatically changed people’s lives (24), but also has had a huge influence on the global economy. It is reported that major economies around the world except China showed negative growth in 2020. Among them, the economic growth rate of the United States, Japan, Germany, France, and Britain was −3.5, −4.8, −5.0, −9.0, and − 10.0%, respectively (25). To prevent and control the transmission of the COVID-19, Chinese government have had to implement a series of lockdown policies, which had led to difficulties in rework, shutdown of work and production, which greatly influenced the demand of employment (26, 27). Affected by the epidemic, global working hours reduced by 5.2% in the first quarter of 2020, and the effect was expanded in the second quarter, reaching 18.2%, then dropped to 7.2 and 4.6% in the third and fourth quarter, respectively (28). The COVID-19 pandemic has caused enormous disruptions in the U.S. labor market. According to the job posting data, the COVID-19 led to a 30% decline in labor demand, in comparison with the same period in 2019 (29). By May 2020, the unemployment rates in Europe and the United States have risen to 7.4 and 15%, respectively (30). The significant increase of the unemployment rate and the resulting reduction of support sources and employment opportunities have become the major features of job (31).

Figure 1 reports the supply and demand situation of college graduates’ employment, and points out the changing trend of employment prosperity index. The CIER index is calculated by using the ratio of the number of recruitment demand to the number of job supply. When the value is greater than 1, it indicates that the job market demand exceeds supply, and the degree of employment prosperity is high. On the contrary, if the value is less than 1, it indicates that the job supply exceeds job demand, and the employment prosperity degree is low (20). In China, as shown in Figure 1, under the influence of the COVID-19, the recruitment demand of enterprises in 2020 showed a trend of first decreasing and then increasing, but after the second quarter of 2021, the recruitment demand declined continuously. At the same time, the employment prosperity index showed a continuous decline trend in the first and second quarters of 2020, then rising in the third and fourth quarters of 2020, and it declined after the second quarter of 2021 (20). Study also indicates that the mismatch between supply and demand in the job market caused by the COVID-19 has a significant influence on the college graduates who are going to walk off the school and walk into the job market (32).

Furthermore, the destruction of the job market induced by the COVID-19 has caused the rise of employment uncertainties and the scarce of substantial amount of high-quality recruitment positions (33). Particularly in tourism, aviation, hotel industry, and those micro, small, and medium-sized enterprises with worse risk-resistance, the recruitment demand declined significantly, and even layoff crisis occurred (34). Besides, tourism, aviation, hotel industry are the major industries that recruit substantial amount of college graduates, which unavoidably result in the decrease of jobs in the labor market for college graduates. However, in the context of evolving economic realities post-COVID-19, with the recovery and transformation of the economy, some emerging industries, such as the digital economy, online education and telemedicine, will usher in rapid growth in recruitment demand. Meanwhile, job opportunities will increase when external demand shifts to domestic demand, as China begins to speed up the construction of new infrastructure in the fields of 5G networks, internet of things, big data, artificial intelligence, industrial internet and smart cities. Consequently, the recruitment demand for college graduates in these emerging industries will increase. Although these emerging industries are developing rapidly, the growth rate of their employment demand cannot fully compensate for the decline of recruitment demand in industries such as tourism, aviation, and hotels. Therefore, the recruitment demand for college graduates has reduced (35), which leads to college graduates’ employment difficulty.

### 3.2 Employment competition rise

**Figure 5:**
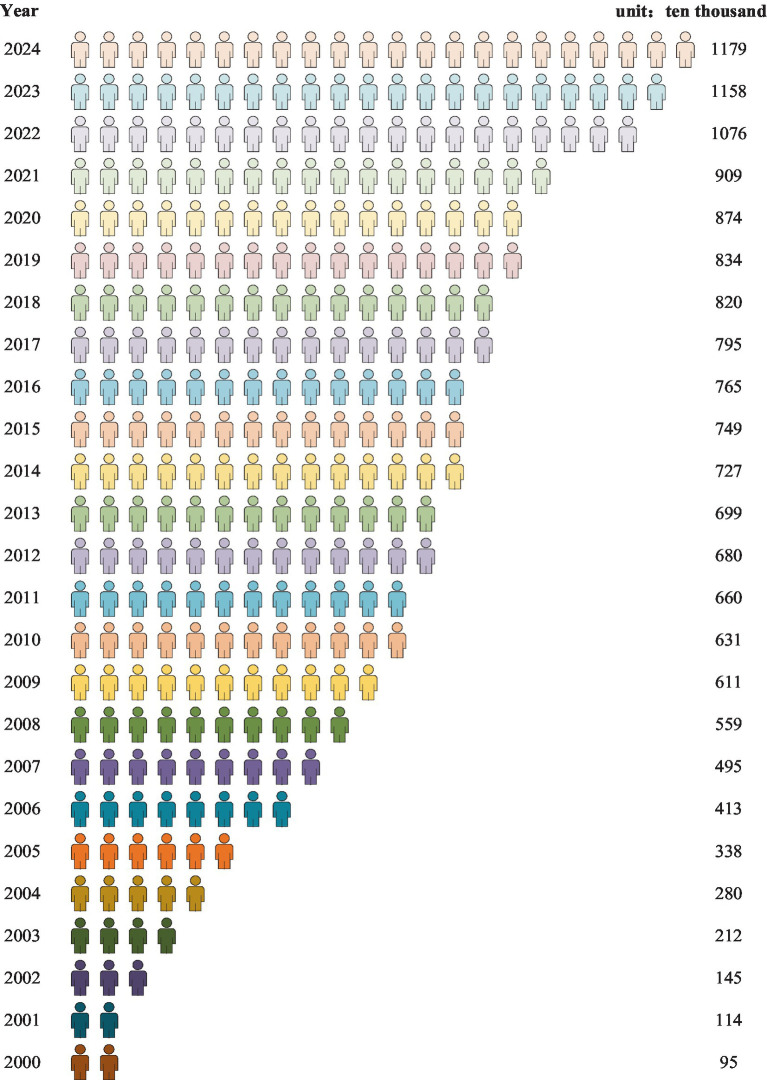
The number of college graduates in China from 2000 to 2024. Data source: collected from Ministry of Education of the People’s Republic of China.

Since Chinese colleges and universities expanded enrollment in 1999, the number of college graduates has increased year after year, as shown in Figure 5. The number of college graduates in China in 2020 is 8.74 million, in 2021 is 9.09 million, in 2022 is 10.76 million, in 2023 is 11.58 million and in 2024 is 11.79 million (Figure 5), respectively, which inevitable leads to a large pressure of concentrated employment and a fierce employment competition (20). Second, the mobility of international students leads to increased domestic employment competition. The COVID-19 epidemic has changed the situation of world higher education, particularly the mobility of international students (36). On the one hand, to tackle the COVID-19 pandemic, countries across the world have implemented a range of lockdown policies, resulting in restrictions on cross-border mobility for international students (37). Therefore, the number of domestic students choose to study abroad has been significantly decreased, and a large number of students have chosen to continue their studies in China rather than to study abroad (38). This inevitably increases the pressure of domestic college graduates to pursue further education in China, which is one of the main form of employment. On the other hand, due to the COVID-19, the global economic recession continued to spread and further resulted in a fierce job competition. An increasing number of Chinese students studying abroad chose to return to China to continue their careers. Research has shown that the number of overseas-educated graduates return to China to seek job opportunities in 2020 increased by 67.3% year on year due to the COVID-19 (39). The surge quantity of returned oversea talents has resulted in a fierce domestic job competition and a deterioration employment environment (40). This has further squeezed the employment opportunities of domestic college graduates and narrowed the employment space, which leads to college graduates’ employment difficulty (41). Although some emerging industries have risen rapidly in the context of evolving economic realities post-COVID-19, new employment opportunities have not fully filled the job vacancies caused by the epidemic. The adjustment of this industry structure has made the supply and demand relationship in the labor market more tense, intensifying employment competition of college graduates.

### 3.3 Employment channels change

Before the outbreak of the epidemic, large-scale offline recruitment fairs were the main channels for college graduates to apply for jobs. Colleges and universities hold offline campus recruitment fairs to improve mutual understanding between employers and college graduates and to achieve two-way selections between employers and college graduates (41). However, the COVID-19 epidemic has hindered the job search channels of college graduates (42). During the epidemic prevention and control period, to reduce personnel gathering, offline recruitment has been cancelled. To minimize the impact of the COVID-19 epidemic on the employment, many foreign recruitment agencies were forced to launch an online recruitment to recruit new talent (43–46). In China, because of the COVID-19, the recruitment form is also forced transform from the previous offline recruitment mode into the online mode, namely, the “cloud recruitment” (47). As shown in Figure 2, from 2019 to 2023, the main channels for college graduates to apply for jobs were recruitment websites and enterprise official websites. The proportion of college graduates seeking jobs through offline job fairs and recruitment sessions showed an overall downward trend. And the proportion of college graduates seeking jobs through HR mailbox and internal recommendations was relatively few. However, online recruitment brings great challenges to a large number of college graduates. For example, the online recruitment not only hinders mutual understanding between employers and college graduates, but also affects the efficiency of recruitment by employers and the assessment of comprehensive qualities such as communication and practical abilities of college graduates (48). Moreover, college graduates can only search for jobs online due to the epidemic, which undoubtedly leads to prolonged job search and further increase the employment difficulty (49, 50). However, in the context of evolving economic realities post-COVID-19, the emergence of emerging online recruitment platforms, social media, and various recruitment apps have provided college graduates with more job search options.

### 3.4 Psychological anxiety increase

**Figure 6:**
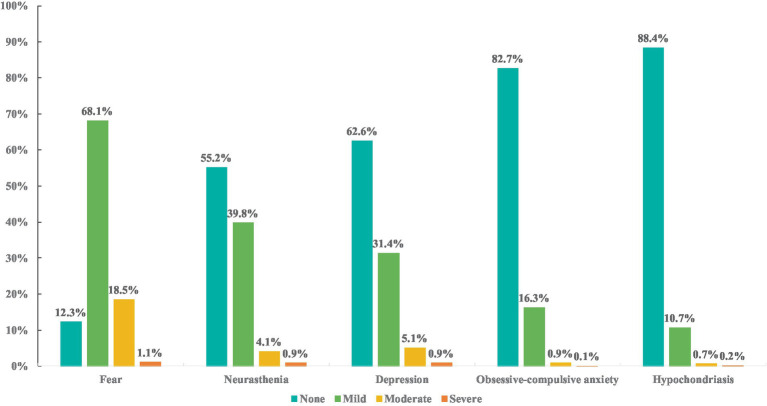
The symptom grading of college students’ PQEEPH in various dimensions during the COVID-19 pandemic. Data source: the result from survey in Huang et al. (59).

Many studies have found that during COVID-19, anxiety has been the biggest problem across the world, followed by sleeping problems, and depression (51, 52). Existing studies suggest that the COVID-19 has a significant impact on the mental health of college graduates around the world (53, 54). It is reported that the outbreak of COVID-19 has caused a high detection rate of anxiety (27.5%) and depression (16.1%) symptoms among college students in the United States (55) and France (56). Furthermore, a cross-country research of nine countries including Poland, Russia, and Germany also confirmed this conclusion (anxiety: 30%, depression: 40.3%) (57). In response to the severe epidemic, the Chinese government adopted lockdown policy to prevent and control viral transmissions. During the COVID-19 lockdown period, people were confronted with anger, boredom, and loneliness, which increased psychological problems such as depression, stress, and anxiety. Studies have shown that COVID-19 home confinement created various psychological impacts, which has a negative effect on the emotional state caused by depression and anxiety (2, 58). In China, during the COVID-19 epidemic, the emotional responses of college students, from the most serious to the least, were fear, neurasthenia, depression, obsessive-compulsive anxiety, and hypochondriasis, with the incidences being 87.7% (2,648/3,019), 44.8% (1,353/3,019), 37.4% (1,129/3,019), 17.3% (522/3,019), and 11.6% (350/3,019), respectively (Figure 6) (59). Although the global epidemic is controlled, the economy of China has gradually resumed and developed, but the negative influence of the epidemic on college graduates’ psychological health and employment continues (60). The COVID-19 pandemic and the economy recession not only made the employment situation of college graduates more serious, but also increased the uncertainty of job seeking (61). The phenomenon of staff reduction and bankrupt of enterprises happens constantly, which reduced labor demand (62). Moreover, due to the COVID-19, the salary has decreased, the supply of well-paid jobs has declined, and the job competition has intensified (63), causing the employment anxiety, which has a great negative influence on the employment mentality and employment status of college graduates. Studies have shown that the increasing anxiety caused by the epidemic places a serious psychological burden on college graduates, making it hard for them to find a suitable job (64).

Second, college graduates are confronted with issues, such as graduation projects, graduation internship, graduation thesis, graduation defense, postgraduate entrance examination and so on. However, various arrangements have been delayed again and again due to the COVID-19, including the graduation project is short of the essential experimental device or materials, the graduation dissertation lacks personally direction of the instructor and the graduation practice cannot be finished timely (41). All of the above factors have affected graduation defense and employment of college graduates. In this situation, college graduates are inclined to be anxious, confused, and fear, which also has a negative impact on the job employment (65). Research has shown that the stronger the anxiety of the epidemic, the stronger the employment crisis sense and the weaker the confidence in job hunting, which affected their probability of successful employment (62).

Third, most college graduates who graduated during the outbreak of the COVID-19 were mainly “post-95 s” or “post-00s.” Most of them were lived in a comparatively advantageous conditions, lacked hard working spirit and the experience in resistance to frustration and shock (41). Furthermore, college graduates are facing a particular development stage and are easily inclined to be anxious and confused (66, 67).

Fourth, in the context of evolving economic realities post-COVID-19, with the recovery and transformation of the economy, the appearance of some emerging industries has provided new employment opportunities for college graduates. However, these emerging industries often require college graduates to possess higher skills and qualities, which college graduates cannot meet the need of the market, leading to their employment anxiety.

### 3.5 Employment structural contradiction intensify

In recent years, the mismatch between the skills owned by college graduates and the skills needed by jobs, which is quite common and is one of the major reason for job difficulty among college graduates in China (68–70). The structural contradiction in employment caused by the COVID-19 epidemic continues to affect the U.S. labor market (71). On the one hand, many college graduates are unable to meet the need of the ideal enterprise with good treatment and good conditions. On the other hand, most of the available job openings are the unstable and lower-wage ones that the college graduates avoid choose. The result is that employers cannot recruit employees and college graduates cannot find jobs. The structural contradiction between employment difficulty and recruitment difficulty highlights the disharmony between the structure of higher education and the demand for talents of economic and social development (72). Moreover, during the COVID-19 epidemic, the structural mismatch between the skills owned by college graduates and the skills needed by jobs becomes the major challenge for college graduates’ employment in China (73). In addition, research has shown that the employment structural contradiction was more provident and become the main contradiction in the employment field under the impact of the COVID-19 (20). As shown in Figure 3, from the first quarter of 2018 to the third quarter of 2022, the disparity between CIER index for college graduates and the national employment market CIER index continued to widen, rising from 0.17 in the first quarter of 2018 to 1.06 in the third quarter of 2022, showing an overall upward trend, indicating that the structural contradiction in China’s employment market has been intensifying.

Furthermore, in the context of evolving economic realities post-COVID-19, the global economy is facing a trend of decreasing increments and weakening momentum. China is also entering an era characterized by strategic opportunities coexisting with challenges and risks. The “World Employment and Social Outlook: Trends 2024” report released by the International Labor Organization predicts that in 2024, the global workforce is expected to increase by 2 million, leading to a rebound in the global unemployment rate from 5.1% in 2023 to 5.2% in 2024 (74). While the global unemployment rate rebounds in 2024, the labor shortages in some specific industries continue to widen. This contradiction reflects the reality that the supply and demand relationship in the global labor market is difficult to restore balance in the short term. The rebound in unemployment rate coexists with labor shortages, further exacerbating global employment structural contradictions. The employment situation of college graduates remains grim. Systemic reform of the global labor market is urgently needed.

## 4 Countermeasures taken by Chinese government and colleges and universities in respond to the impacts on the employment of college graduates caused by the COVID-19

**Figure 7:**
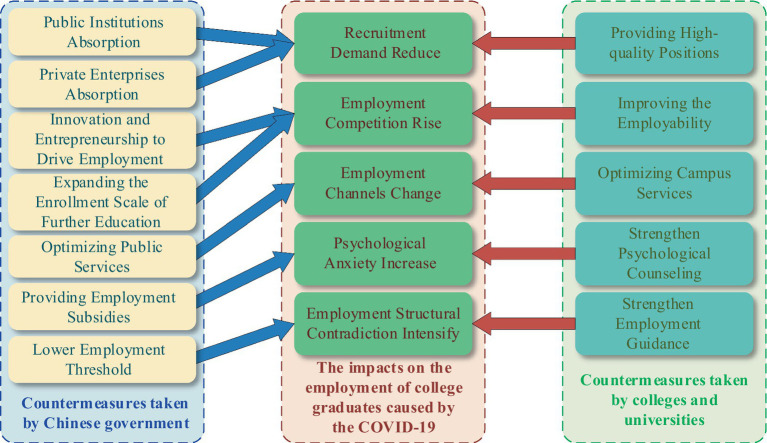
Countermeasures taken by Chinese government and colleges and universities to promote the better and fuller employment of college graduates.

At present, with effects of world economic recession, domestic economy downlink and the COVID-19, college graduates are facing a more and more serious employment situation. Based on the severe situation of college graduates’ employment, president Xi Jinping gave a series of important instructions and emphasized that “We should pay more attention to the employment of college graduates. We must jointly do a good work of the graduation, employment and examination and recruitment of the college graduates, to ensure that they can graduate smoothly and obtain employment as soon as possible.” As shown in Figure 7, in respond to the five negative impacts of the COVID-19 on the employment of college graduates, Chinese government has implemented seven employment promotion measures while colleges and universities have introduced five employment promotion measures to jointly promote the better and fuller employment of college graduates.

### 4.1 Countermeasures taken by Chinese government in respond to the impacts on the employment of college graduates caused by the COVID-19

**Figure 8:**
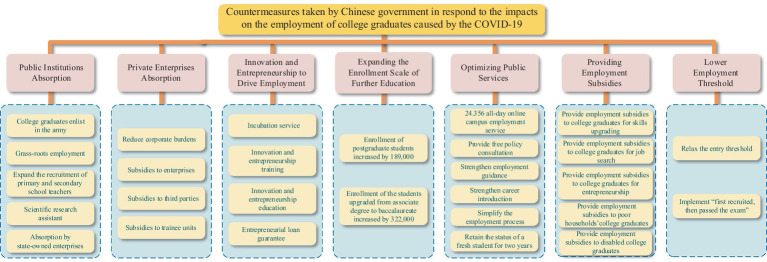
Countermeasures taken by Chinese government in respond to the impacts on the employment of college graduates caused by the COVID-19.

As shown in Figure 8, in response to the pandemic, Chinese government has implemented a wide range of specific employment promotion measures to promote the employment of college graduates.

#### 4.1.1 Public institutions absorption

The government pays great attention on strengthen the exemplary leading role of government departments and state-owned enterprises and expands the job supply of state-owned enterprises and public institutions for college graduates through flexible and ingenious job creation (75). In a word, the government facilitated college graduates to the local employment required by the country mainly through the following five ways: guiding college graduates to join the army, encouraging college graduates to work at the grassroots units, expanding the recruitment of primary and secondary school teachers, increasing research assistant positions and attracting college graduates in state-owned enterprises (24).

The employment policy at the grass-roots units combines the employment requirement of college graduates with the aim of all-round well-off society construction, guides more college graduates to work at the grass-roots unit in remote areas, which effectively promotes talent mobility and regional balanced development and alleviates the employment pressure caused by the epidemic. In addition, enrolling college graduates into army is one of the significant methods to facilitate the high-quality development of the military and strengthen the national defence force (24).

#### 4.1.2 Private enterprises absorption

The government has taken measures to expand the demand of recruitment to promote the employment of college graduates, for instance, stabilizing the job positions of small and medium enterprises by reducing corporate burdens,^3^ including tax reduction and exemption, unemployment insurance refunds, offering employment subsidies and guaranteed loans and interest subsidies (76). The government also subsidized the job training agencies to facilitate internship and employment of college graduates and to enhance the college graduates’ ability of getting employed (24).

#### 4.1.3 Innovation and entrepreneurship to drive employment

Since the scale and increment of college graduates have reached a high record in recent years, innovation and entrepreneurship plays a crucial role in driving employment and economic growth. Therefore, Chinese government issued many business measures to encourage and help college graduates to build up their own businesses (20). The government introduced a series of policies to boost mass entrepreneurship and innovation, including incubation service, education and training of innovation and entrepreneurship, entrepreneurship guarantee loans (24).

#### 4.1.4 Expanding the enrollment scale of further education

The government not only enlarged the enrollment scale of postgraduate students but also expanded the enrollment scale of the students upgraded from associate degree to baccalaureate (10) to reduce the number of fresh graduates and alleviate the employment pressure caused by the COVID-19. According to the Ministry of Education News, in 2020, the enrollment of postgraduate students increased by 189,000 while the enrollment scale of the students upgraded from associate degree to baccalaureate increased by 322,000.^4^

#### 4.1.5 Optimizing public services

To promote efficient employment of college graduates, Chinese government has worked on various aspects, including broadening employment channels, upgrading employment policies, optimizing employment services and strengthening precise matching of information between college graduates and employers. Furthermore, the Ministry of Education, together with twelve social recruitment agencies and colleges and universities, has launched a 24.365 all-day online campus recruitment service platform, which providing continuous campus recruitment services for college graduates (10). In addition, the government also has provided free employment policy consultation, career guidance, job recommendation, and other employment services for college graduates, such as simplifying the recruitment process, retaining the status of unemployed graduates for 2 years (24).

#### 4.1.6 Providing employment subsidies

The government increased financial subsidies to college graduates to guarantee their basic livelihood and make sure that college graduates can concentrate on seeking jobs. The types of subsidies for college graduates consisted of personal skills enhancement subsidies, job seeking and entrepreneurship subsidies (24). Furthermore, for college graduates with employment difficulties, for instance, poor households’ college graduates and disabled college graduates, the government also distributes employment subsidies to help them to get through the hard times.

#### 4.1.7 Lower employment threshold

To invigorate the job market and stimulate development and social creativity, the government has dynamically optimized the National Vocational Qualification Catalogue and lowered employment threshold for college graduates who failed to gain the professional qualification certificate on schedule due to the COVID-19 epidemic. Due to the impact of the COVID-19, the relevant qualification certificates examinations could not be conducted on time. In September 2020, the Ministry of Education proposed a policy to reform the certification of primary and secondary school teachers, lowering the threshold for qualified students to enter the teaching profession.^5^ The government introduced the policy that the teachers were approved to obtain the teaching certificate after inauguration, so that it could promote the smooth employment of college graduates (24).

### 4.2 Countermeasures taken by colleges and universities in respond to the impacts on the employment of college graduates caused by the COVID-19

**Figure 9:**
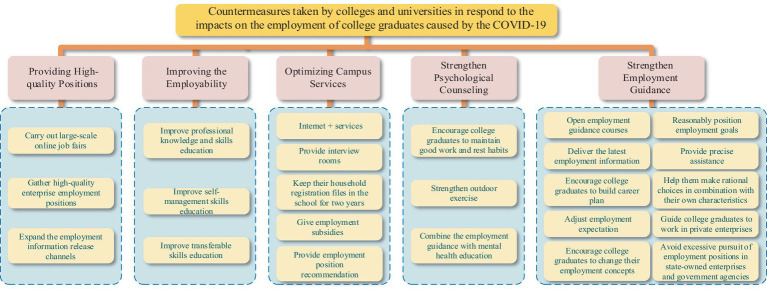
Countermeasures taken by colleges and universities in respond to the impacts on the employment of college graduates caused by the COVID-19.

As shown in Figure 9, in response to the pandemic, Chinese colleges and universities also have introduced a series of specific employment promotion measures to boost the employment of college graduates.

#### 4.2.1 Providing high-quality positions

Colleges and enterprises have to cancel offline campus recruitment fairs due to the impact of the COVID-19, which are the main channel for college graduates to find a job (10). However, colleges and universities responded quickly and carried out large-scale online job fairs to provide high-quality positions for college graduates. Furthermore, in order to provide more high-quality positions for college graduates, colleges and universities actively connected with enterprises, fully gathered high-quality employment positions, and invited enterprises to enter the campus to recruit college graduates through the combination of online and offline (20). In addition, colleges and universities not only taken full advantage of Internet technology, but also expanded the employment information release channels for college graduates, such as the job hunting information website, WeChat official account and employment QQ group.

#### 4.2.2 Improving the employability

As we all know, whether college graduates can achieve high-quality employment mainly depends on their personal employability (77). A previous study noted that colleges and universities should concentrate on enhancing the key employability of college graduates, so that college graduates can adapt and get involved in work as soon as possible (78). To promote the employment of college graduates, colleges and universities have introduced multiple methods to enhance the employability of college graduates, such as taken classroom teaching as the primary way for professional knowledge and skills education, taken daily management as the carrier for self-management skills education and taken social practice as the platform for transferable skills education (79).

#### 4.2.3 Optimizing campus services

To enhance the employment and entrepreneurship service for college graduates, colleges and universities built the “Internet + services” smart employment system, which greatly simplified the employment process of college graduates. College graduates can complete the entire employment process in one time through the “Internet + services” smart employment system, including sign employment agreements with enterprises (80), print the employment agreement on the campus self-service terminal and stamp the employment agreement. Second, colleges and universities also provided interview rooms for college graduates to apply for a job. In addition, colleges and universities improved the employment assistance for college graduates, such as keeping their hukou and files in the school for 2 years, giving employment subsidies, providing employment position recommendation and so on.

#### 4.2.4 Strengthen psychological counseling

Because of the COVID-19, college graduates are inclined to be worried and anxious in job seeking (81). Moreover, it is reported that epidemic anxiety has a negative effect on college graduates’ employment confidence (62). Therefore, university instructors, counselors, employment guiding departments and psychological consulting centers jointly enhanced college graduates’ employment confidence and encouraged college graduates to maintain good work and rest habits, enhance outdoor activities (82), and alleviate anxiety amid the epidemic (60, 83). To alleviate college graduates’ mental stress caused by the COVID-19, colleges and universities also combined the career instruction with psychological health education and attached great importance to the psychological health problems of college graduates (84).

#### 4.2.5 Strengthen employment guidance

In response to the serious employment situation caused by the COVID-19, colleges and universities enhanced the employment guidance for college graduates, such as opening employment guidance courses, delivering the latest recruitment information, providing precise assistance, guiding college graduates to develop a job search plan, encouraging college graduates to adjust their employment ideas according to changes, and helping them to combine their interests and self-expertise with career choices and choose career development paths more rationally (20). In addition, since micro, small, and medium-sized enterprises are the main forces to absorb the employment of college graduates. Colleges and universities also encourage college graduates to search for jobs in private companies and avoid only seeking jobs in state-owned enterprises and government institutions (85).

## 5 Implications

In the post-epidemic period, the employment situation of college graduates remains severe, government and colleges and universities still need to make every effort to help college graduates and strengthen the sustainability and persistence of the employment promotion measures for college graduates. Furthermore, government and colleges and universities also should improve and optimize the existing employment measures and roll out a new series of measures to promote the employment of college graduates. Therefore, this paper provides the following implications for the government and colleges and universities.

### 5.1 Implications for the government

(1) In terms of improving the public institutions absorption measures, the government should steadily increase the recruitment number of public institutions and arrange the exams of public institutions’ recruitment as early as possible to leave college graduates more time for job seeking.

(2) In terms of improving the private enterprises absorption measures, the government should not only collaborate with leading enterprises in various fields to recommend large enterprises to the online recruitment platform, but also drive more micro, small, and medium-sized enterprises to register on the online recruitment platform.

(3) In terms of improving the innovation and entrepreneurship to drive employment measures, the government should combine regional characteristics with economic development to promote the development of innovation and entrepreneurship.

(4) In terms of improving the expanding the enrollment scale of further education measures, the government should anticipate that the college graduates choose to further education will confront employment pressure again within several years. As a consequence, in response to the “depreciation of academic qualifications,” it is necessary for the government to optimize economic structure and achieve industrial upgrading as quickly as possible and create more employment positions that recruit high-end talents.

(5) In terms of improving the providing employment subsidies measures, the government should strengthen supervision and guarantee that relevant departments and enterprises carry out policies, so that college graduates can indeed enjoy various subsidy policies.

(6) In terms of improving the optimizing public services measures, the government should innovate supply–demand matching employment mechanism, further deepen the integration of industry and education and school-enterprise cooperation, better integrate enterprise resources, and provide more adequate employment opportunities and high-quality services for college graduates.

(7) In terms of improving the lower employment threshold measures, the government should further reduce the number and type of vocational qualifications, promote the identification of socialized vocational skill grade, and reasonably relax or cancel work experience requirements for taking exams for some vocational qualifications.

### 5.2 Implications for colleges and universities

(1) In terms of improving the providing high-quality positions measures, colleges and universities not only should broaden market-oriented employment channels to provide college graduates with more high-quality job information, but also encourage them to take full advantage of the online recruitment platforms.

(2) In terms of improving the improving the employability measures, colleges and universities should closely keep up with the development trend of emerging industries, continue to adjust the student cultivation plan and program and optimize teaching contents and course system, so that talent cultivation can closely connect with the demands of the job market.

(3) In terms of improving the strengthen employment guidance measures, colleges and universities should carry out more precise employment guidance, internships and skill training projects that related to core skills and make full use of modern information technology to provide college graduates with personalized and convenient employment guidance.

(4) In terms of improving the optimizing campus services measures, colleges and universities not only should provide services for the fresh college graduates, but also should provide continuous services for the graduates of previous years.

(5) In terms of improving the strengthen psychological counseling measures, colleges and universities should comprehensively grasp the dynamic psychological changes of college graduates and prepare emergency plan.

## 6 Conclusion

As shown in Figure 4, the number of college graduates in China in 2019 is 8.34 million, in 2020 is 8.74 million, in 2021 is 9.09 million, in 2022 is 10.76 million, in 2023 is 11.58 million and in 2024 is 11.79 million, respectively and the number of employed college graduates in China in 2019 is 7.4 million, in 2020 is 7.64 million, in 2021 is 7.82 million, in 2022 is 9.03 million, and in 2023 is 10.03 million, respectively.

The COVID-19 has mainly caused five negative impacts on the employment of college graduates, including recruitment demand reduce, employment competition rise, employment channels change, psychological anxiety increase and employment structural contradiction intensify. Furthermore, under the background of world economic recession and domestic economy downlink, college graduates are facing serious employment challenge. In addition, the number of college graduates has increased year by year. In respond to the five negative impacts of the COVID-19 on the employment of college graduates, Chinese government has implemented seven employment promotion measures while colleges and universities have introduced five employment promotion measures to jointly promote the better and fuller employment of college graduates. With joint efforts from Chinese government and colleges and universities, the number of employed college graduates in China from 2019 to 2023 has increased year by year, which suggest that countermeasures taken by Chinese government and colleges and universities have effectively promoted the employment of college graduates to a large extent.

In the post-epidemic period, the employment status of college graduates is still severe. Based on the findings of this study, this paper makes suggestions for the government and colleges and universities to promote the employment of college graduates. First, the government should continue to promote the action plan of “expanding jobs and promoting employment in the graduation season.” Second, the government and colleges and universities should not only continuously improve and optimize the existing employment promotion measures to promote the employment of college graduates, but also strengthen supervision and guarantee that relevant departments and enterprises carry out employment promotion measures, so that college graduates can indeed benefit from these employment promotion measures. Third, in the background of the intensified employment structural contradiction in the college graduates’ job market, colleges and universities should strengthen employment guidance, encourage college graduates to change their employment concepts timely and adjust their employment expectations reasonably in combination with the employment status and enterprise needs, make accurate and targeted recommendations according to their job-hunting wishes, and help them find a suitable career development path. Fourth, in the background of the technological revolution and industrial upgrading, the government should promote the integration of industry and education and cooperation between colleges and enterprises, and encourage more college graduates to receive vocational and technical education. The findings of this study will make references for government and colleges and universities worldwide to help college graduates overcome the difficult employment season under the persist impact of the COVID-19 epidemic and other similar emergency situations.

However, this paper still had the following limitations, which need further study. Considering the evolving nature of the pandemic and its long-term impacts on the global economy and employment landscape, on the one hand, we will explore the changing trends in the employment market for college graduates, the development trends of emerging industries, and the structural changes in the labor market. Through long-term tracking and analysis of data, we will further reveal the profound impact of the epidemic on the employment landscape, providing more accurate and cutting-edge employment guidance for college graduates. On the other hand, we will explore effective measures for systemic reform of the global labor market, conduct targeted research, summarize more effective employment promotion measures, and give corresponding suggestions to solve the problem of the rebound of unemployment rate and the coexistence of labor shortage, and to alleviate structural employment contradictions worldwide.

## Author contributions

HW: Conceptualization, Data curation, Formal analysis, Funding acquisition, Investigation, Methodology, Project administration, Resources, Software, Supervision, Validation, Visualization, Writing – original draft, Writing – review & editing. CW: Data curation, Writing – review & editing.

## Footnotes

^1^Source: Ministry of Education News. http://www.moe.gov.cn/jyb_zzjg/huodong/202312/t20231205_1093287.html

^2^Source: Ministry of Education News. http://www.moe.gov.cn/srcsite/A15/s3265/202312/t20231205_1093196.html?from=timeline&isappinstalled=0

^3^Source: Ministry of Human Resources and Social Security News. http://www.mohrss.gov.cn/SYrlzyhshbzb/dongtaixinwen/buneiyaowen/202003/t20200326_363672.html.

^4^Source: Ministry of Education News. https://www.gov.cn/zhengce/2020-02/29/content_5484774.htm.

^5^Source: Ministry of Education News. http://en.moe.gov.cn/features/2021TeachersDay/Educators/202109/t20210922_565560.html

## References

[1] Younas M Noor U Zhou X Menhas R Qingyu X. COVID-19, students satisfaction about e-learning and academic achievement: mediating analysis of online influencing factors. Front Psychol. (2022) 13:948061. doi: 10.3389/fpsyg.2022.948061, PMID: 36081717 PMC9444837

[2] Sang X Menhas R Saqib ZA Mahmood S Weng Y Khurshid S. The psychological impacts of COVID-19 home confinement and physical activity: a structural equation model analysis. Front Psychol. (2021) 11:614770. doi: 10.3389/fpsyg.2020.614770, PMID: 33519638 PMC7843378

[3] Asante LA Mills RO. Exploring the socio-economic impact of COVID-19 pandemic in marketplaces in urban Ghana. Afr Spectr. (2020) 55:170–81. doi: 10.1177/0002039720943612

[4] Chen T Peng L Yin X Jing B Yang J Cong G. A policy category analysis model for tourism promotion in China during the COVID-19 pandemic based on data mining and binary regression. Risk Manag Healthcare Policy. (2020) 13:3211–33. doi: 10.2147/RMHP.S284564, PMID: 33408543 PMC7781111

[5] Fu P Jing B Chen T Xu C Yang J Cong G. Propagation model of panic buying under the sudden epidemic. Front Public Health. (2021) 9:675687. doi: 10.3389/fpubh.2021.675687, PMID: 33968890 PMC8100230

[6] Natali D. COVID-19 and the opportunity to change the neoliberal agenda: evidence from socio-employment policy responses across Europe. Transfer. (2022) 28:15–30. doi: 10.1177/10242589221097231

[7] Dewi NI Melati FC. The impact on economic and environmental development of COVID-19 pandemic: a case study in Indonesia. Ekuilibrium. (2021) 16:1–11. doi: 10.24269/ekuilibrium.v16i1.2021.pp1-11

[8] Ferreira JP Ramos P Barata E Court C Cruz L. The impact of COVID-19 on global value chains: disruption in nonessential goods production. Region Sci Policy Pract. (2021) 13:32–54. doi: 10.1111/rsp3.12416, PMID: 38607863 PMC8250671

[9] Karn SK. Impact of COVID-19 on Nepalese economy. Int J Soc Sci Manage. (2021) 8:348–51. doi: 10.3126/ijssm.v8i2.36637

[10] Chen T Rong J Peng L Yang J Cong G Fang J. Analysis of social effects on employment promotion policies for college graduates based on data mining for online use review in China during the COVID-19 pandemic. Healthcare. (2021) 9:846. doi: 10.3390/healthcare9070846, PMID: 34356224 PMC8307509

[11] Zhang GW Wu T. The impact of the COVID-19 outbreak on employment in China. Chin J Popul Sci. (2020) 126:11–20.

[12] Gao BQ. Exploration on employment practice of college graduates under the background of epidemic prevention and control. J Anyang Normal Univ. (2020) 6:55–7. doi: 10.16140/j.cnki.1671-5330.2020.06.012

[13] Li J Matlay H. Graduate employment and small businesses in China. Industry High Educ. (2005) 19:45–54. doi: 10.5367/0000000053123637

[14] Ogunlel YI. Impact of the programmes of the National Directorate of employment on graduate employment and unemployment in Kaduna state of Nigeria. Pak J Soc Sci. (2012) 9:40–5. doi: 10.3923/pjssci.2012.40.45

[15] Poon PL Man FL Tang SF. The impact of industrial placement on BIS graduate employment and further educational advancement. Inform Syst Educ J. (2021) 19:4–12.

[16] Mo R Chen Y Bao CL Huang XM. The impact of the COVID-19 outbreak, SARS epidemic and the global financial crisis on employment and comparative analysis of their responses. China Labor. (2020) 1:16–30. doi: 10.19390/j.cnki.chinalabor.2020.01.002

[17] Li X Pu R Phakdeephirot N. The influence of achievement motivation on college students’ employability: a chain mediation analysis of self-efficacy and academic performance. Front Psychol. (2022) 13:972910. doi: 10.3389/fpsyg.2022.972910, PMID: 36275301 PMC9582943

[18] Luo X. Research on employment pressure and countermeasures of college graduates after the epidemic. In 2021 2nd Asia-Pacific conference on image processing, electronics and computers. (2021) (pp. 202–205).

[19] Song HT Ge CH Chang LX Zhao TT Zhang XL. Investigation on the psychological status of college students during the coronavirus disease-2019 epidemic. J Gen Psychol. (2021) 149:1–12. doi: 10.1080/00221309.2021.1893637 33709883

[20] Mao Y Zhang Y Bai J Zhang L Hu W. The impact of COVID-19 on the employment status and psychological expectations of college graduates: empirical evidence from the survey data of Chinese recruitment websites. Front Psychol. (2022) 13:1039945. doi: 10.3389/fpsyg.2022.1039945, PMID: 36438406 PMC9692114

[21] Sven J. Nordic employment policies–change and continuity before and during the financial crisis. Soc Policy Adm. (2011) 45:131–45. doi: 10.1111/j.1467-9515.2010.00760.x

[22] Méndez R Sánchez-Moral S Malfeito-Gaviro J. Employment changes in knowledge-based industries in large urban areas of Spain: impact of the economic crisis and austerity policies. Environ Plan C. (2016) 34:963–80. doi: 10.1177/0263774X15614698

[23] Mok KH. Massification of higher education, graduate employment and social mobility in the greater China region. Br J Sociol Educ. (2016) 37:51–71. doi: 10.1080/01425692.2015.1111751

[24] Wu M Hao X Tian Y. Employment management policies for college graduates under COVID-19 in China: diffusion characteristics and core issues. Healthcare. (2022) 10:955. doi: 10.3390/healthcare10050955, PMID: 35628092 PMC9140726

[25] Jiang D Wang X Zhao R. Analysis on the economic recovery in the post-COVID-19 era: evidence from China. Front Public Health. (2022) 9:787190. doi: 10.3389/fpubh.2021.787190, PMID: 35141186 PMC8818724

[26] Gao WS. The impact of COVID-19 pandemic on China’s employment and the countermeasures to it. J Grad Sch Chin Acad Soc Sci. (2020) 3:21–31.

[27] Mo R Chen Y Bao CL Huang XM. Comparative analysis of the effects of COVID-19 epidemic and SARS epidemic and international financial crisis on employment and countermeasures. China Labor. (2020) 1:16–30. doi: 10.19390/j.cnki.chinalabor.2020.01.002

[28] Mosbah EB Dharmapala PS. Evaluating the effects of COVID-19 and vaccination on employment behaviour: a panel data analysis across the world. Sustain For. (2022) 14:9675. doi: 10.3390/su14159675

[29] Shuai X Chmura C Stinchcomb J. COVID-19, labor demand, and government responses: evidence from job posting data. Bus Econ. (2021) 56:29–42. doi: 10.1057/s11369-020-00192-2, PMID: 33311717 PMC7719850

[30] Crooton Blog. The impact of COVID-19 on the recruitment industry. (2020). Available at: https://www.crooton.com/2020/12/18/the-impact-of-covid-19-on-the-recruitment-industry/

[31] Blustein DL Guarino PA. Work and unemployment in the time of COVID-19: the existential experience of loss and fear. J Humanist Psychol. (2020) 60:702–9. doi: 10.1177/0022167820934229

[32] Shahriar MS Islam KM Zayed NM Hasan K Raisa TS. The impact of COVID-19 on Bangladesh's economy: a focus on graduate employability. J Asian Finance Econ Bus. (2021) 8:1395–403. doi: 10.13106/JAFEB.2021.VOL8.NO3.1395

[33] Moen P Pedtke JH Flood S. Disparate disruptions: intersectional COVID-19 employment effects by age, gender, education, and race/ethnicity. Work Aging Retire. (2020) 6:207–28. doi: 10.1093/workar/waaa013, PMID: 33214905 PMC7543645

[34] Borjas GJ Cassidy H. The adverse effect of the COVID-19 labor market shock on immigrant employment. Soc Sci Electr Pub. (2020) 50:327–67. doi: 10.2139/ssrn.3608526

[35] Mamo WB Feyisa HL Yitayaw MK Tereda SN. Employment status during the COVID-19 pandemic: evidence from Ethiopia. Indian J Labour Econ. (2022) 65:123–35. doi: 10.1007/s41027-022-00365-x, PMID: 35462636 PMC9017086

[36] Mok KH Xiong W Ke G Cheung JOW. Impact of COVID-19 pandemic on international higher education and student mobility: student perspectives from mainland China and Hong Kong. Int J Educ Res. (2021) 105:101718. doi: 10.1016/j.ijer.2020.101718 35719275 PMC9188844

[37] Dai J Sang X Menhas R Xu X Khurshid S Mahmood S. The influence of COVID-19 pandemic on physical health–psychological health, physical activity, and overall well-being: the mediating role of emotional regulation. Front Psychol. (2021) 12:667461. doi: 10.3389/fpsyg.2021.667461 34484032 PMC8415626

[38] Marginson S. Global HE as we know it has forever changed. Times. High Educ. (2020) 26. Available at: https://www.timeshighereducation.com/blog/global-he-we-know-it-has-forever-changed

[39] Zhang K Zeng N Zhang K. Remain or return? An empirical study of influencing factors on the return of Chinese international students during the COVID-19 pandemic. Front Psychol. (2022) 13:1067184. doi: 10.3389/fpsyg.2022.1067184, PMID: 36506958 PMC9727143

[40] Li H Wu Y Qiu WS Gan L. The impacts of COVID-19 on China’s small and medium-size enterprises: a progress report. Bull Natl Nat Sci Found China. (2020) 65:747–52. doi: 10.1016/j.scib.2019.12.021, PMID: 36659108

[41] Ren W. “A study on college graduates’ employment problem in the context of big data based on the event of COVID-19,” in ICBDE’21: 2021 4th international conference on big data and education (Shanghai). (2021) 88–91.

[42] Aucejo EM French J Araya MPU Zafar B. The impact of COVID-19 on student experiences and expectations: evidence from a survey. J Public Econ. (2020) 191, 27392:104271. doi: 10.3386/w27392 32873994 PMC7451187

[43] Selvaraj V Venkatakrishnan S. Role of information Systems in Effective Management of human resources during the COVID-19 pandemic. Systems. (2023) 11:573. doi: 10.3390/systems11120573

[44] Vashistha N Goel A Dhiman A. A study on the impact of COVID-19 pandemic in the recruitment process: with special reference to IT companies of Noida region. Turkish Online J Qualit Inq. (2021) 12:12024–43.

[45] Dillahunt T. R. Israni A. Lu A. J. Cai M. Hsiao J. C. Y Examining the use of online platforms for employment: a survey of U.S. job seekers. In CHI conference on human factors in computing systems. (2021).

[46] Lena H Le Barbanchon T Rathelot R. Job search during the COVID-19 crisis. J Public Econ. (2021) 194:104349. doi: 10.1016/j.jpubeco.2020.104349

[47] Ling H Li J Wang J Chen S. Research on the employment situation of Chinese college students in the prevention and control of major public health events. Psychology. (2021) 12:1230–45. doi: 10.4236/psych.2021.128077

[48] Huang LP. Study on the employment of college graduates under the COVID-19 epidemic-based on Guangdong empirical investigation. Youth Res. (2020) 3:85–95.

[49] Chen YH Zhang Z. The impact of COVID-19 epidemic awareness on employment expectation. J South China Agri Univ Soci Sci Edi. (2020) 19:105–19. doi: 10.7671/j.issn.1672-0202.2020.04.010

[50] Li C. College graduate employment under the impact of COVID-19: employment pressure, psychological stress and employment choices. Educ Research. (2020) 7:4–16.

[51] Ahmed Laar R Zhang Z Menhas R Zhang L Zhu S Fan X. Impact of coronavirus disease of 2019 vaccine on health and physical activities among physical education students in China. Front Public Health. (2022) 10:889311. doi: 10.3389/fpubh.2022.889311, PMID: 35859772 PMC9289447

[52] Germani A Buratta L Delvecchio E Gizzi G Mazzeschi C. Anxiety severity, perceived risk of COVID-19 and individual functioning in emerging adults facing the pandemic. Front Psychol. (2020) 11:567505. doi: 10.3389/fpsyg.2020.567505, PMID: 33364996 PMC7750437

[53] Menhas R Qin L Saqib ZA Younas M. The association between COVID-19 preventive strategies, virtual reality exercise, use of fitness apps, physical, and psychological health: testing a structural equation moderation model. Front Public Health. (2023) 11:1170645. doi: 10.3389/fpubh.2023.1170645, PMID: 37483921 PMC10358774

[54] Jahan U Al-Sariri SA Al-Wahaibi MK Al-Anburi SI. Impact of COVID-19 anxiety on university students’ job seeking behaviour during the pandemic: an empirical study. J Organ Hum Behav. (2022) 11:27–38.

[55] Kibbey MM Fedorenko EJ Farris SG. Anxiety, depression, and health anxiety in undergraduate students living in initial US outbreak “hotspot” during COVID-19 pandemic. Cogn Behav Ther. (2021) 50:409–21. doi: 10.1080/16506073.2020.1853805, PMID: 33433271

[56] Wathelet M Duhem S Vaiva G Baubet T Habran E Veerapa E. Factors associated with mental health disorders among university students in France confined during the COVID-19 pandemic. JAMA Netw Open. (2020) 3:1–13. doi: 10.1001/jamanetworkopen.2020.25591, PMID: 33095252 PMC7584927

[57] Ochnik D Rogowska AM Ku’snierz C Jakubiak M Schütz A Held MJ. Mental health prevalence and predictors among university students in nine countries during the COVID-19 pandemic: A cross-national study. Scientific Reports. (2021) 11:18644. doi: 10.1038/S41598-021-97697-3, PMID: 34545120 PMC8452732

[58] Younas M Dong Y Menhas R Li X Wang Y Noor U. Alleviating the effects of the COVID-19 pandemic on the physical, psychological health, and wellbeing of students: coping behavior as a mediator. Psychol Res Behav Manag. (2024) 16:5255–70. doi: 10.2147/PRBM.S441395, PMID: 38164325 PMC10758179

[59] Daqing H Yan W Xia H Weizhi L Zhilei S. The impact of the COVID-19 epidemic on the mental health of college students. J Naval Med Univ. (2022) 6:709–14. doi: 10.16781/j.CN31-2187/R.20210897

[60] Li YX Chen S. Mental health literacy:basis of mental health education in schools. J Henan Univ. (2022) 110:155. doi: 10.15991/j.cnki.411028.2022.03.004

[61] Wang ZW Li G. The internal dialectical relation between epidemic prevention and control and economic development based on the perspective of national governance. Econ Probl. (2022) 8:9–15. doi: 10.16011/j.cnki.jjwt.2022.08.016

[62] Zheng S Wu G Zhao J Chen W. Impact of the COVID-19 epidemic anxiety on college students' employment confidence and employment situation perception in China. Front Psychol. (2022) 13:980634. doi: 10.3389/fpsyg.2022.980634 36160584 PMC9501885

[63] Wang Q Cao HY Guo HJ. On the cultivation of humanistic quality of science and engineering college students from the employment dilemma under the impact of the epidemic. Jiangsu Voc Inst Arch Technol. (2022) 22:57–60. doi: 10.19712/j.cnki.jsjyxb.2022.01.018

[64] Gao J Wang F Guo S Hu F. Mental health of nursing students amid coronavirus disease 2019 pandemic. Front Psychol. (2021) 12:699558. doi: 10.3389/fpsyg.2021.699558, PMID: 34475837 PMC8407077

[65] Wang YJ Zeng QD Liu N Li HY Liu HX Cheng M. Construction of the employment mental health scale for disabled college students. China J Health Psychol. (2021) 29:1550–5. doi: 10.13342/j.cnki.cjhp.2021.10.023

[66] Schulenberg JE Sameroff AJ Cicchetti D. The transition to adulthood as a critical juncture in the course of psychopathology and mental health. Dev Psychopathol. (2004) 16:799–806. doi: 10.1017/s0954579404040015 15704815

[67] Kessler RC Berglund P Demler O Jin R Merikangas KR Walters EE. Lifetime prevalence and age-of-onset distributions of DSM-IV disorders in the National Comorbidity Survey Replication. Arch Gen Psychiatry. (2005) 62:593–602. doi: 10.1001/archpsyc.62.6.593, PMID: 15939837

[68] Wang T Zeng XQ. Research on the reason and proposal of college graduates structural unemployment. Educ Econ. (2009) 1:1–4.

[69] Yao X Zhang H Le J. Industrial transition and employment of college graduates. Stud Labor Econ. (2014) 2:34–47.

[70] Zeng XQ. Job seeking of college graduates in employment environment under transition. Econ Res J. (2004) 6:87–95.

[71] Liu M. Structural issues in the U.S. labor market are difficult to alleviate in the short term. (2023). Available at: https://www.thepaper.cn/newsDetail_forward_21956133

[72] Shi Q Ren K. The connotation and influencing factors of employment ability of college students in China: based on the comparison between applied universities and research universities. J East China Normal Univ. (2023) 41:1–12. doi: 10.16382/j.cnki.1000-5560.2023.08.00

[73] Jiang S Guo Y. Reasons for college major-job mismatch and labor market outcomes: evidence from China. China Econ Rev. (2022) 74:101822. doi: 10.1016/j.chieco.2022.101822

[74] International Labour Organization. World employment and social outlook: Trends 2024. Int Lab. (2024) 2024:37–60. doi: 10.1002/wow3.204

[75] Yang S Yang J Yue L Xu J Liu X Li W. Impact of perception reduction of employment opportunities on employment pressure of college students under COVID-19 epidemic–joint moderating effects of employment policy support and job-searching self-efficacy. Front Psychol. (2022) 13:986070. doi: 10.3389/fpsyg.2022.986070, PMID: 36337528 PMC9627308

[76] Zhang H. China's employment stabilization policies in response to the impact of the COVID-19 pandemic. Int J Sociol Soc Policy. (2020) 42:201–9. doi: 10.1108/ijssp-05-2020-0167

[77] Shiyuan Y Jinxiu Y Jingfei X Yuling Z Longhua Y Houjian L. Impact of human capital and social capital on employability of Chinese college students under COVID-19 epidemic-joint moderating effects of perception reduction of employment opportunities and future career clarity. Front Psychol. (2022) 13:1046952. doi: 10.3389/fpsyg.2022.1046952, PMID: 36605287 PMC9809468

[78] Minocha S Hristov D Reynolds M. From graduate employability to employment: policy and practice in UK higher education. Int J Train Dev. (2017) 21:235–48. doi: 10.1111/ijtd.12105

[79] Li H Zheng F Zhang J Guo Z Yang H Ren C. Using employment data from a medical university to examine the current occupation situation of Master's graduates in public health and preventive medicine in China. Front Public Health. (2020) 8:508109. doi: 10.3389/fpubh.2020.508109, PMID: 33425824 PMC7793996

[80] Li X. The path of promoting college students’ employment and entrepreneurship education under the supply side reform. Guide Sci Educ. (2022) 12:156–8. doi: 10.16400/j.cnki.kjdk.2022.12.051

[81] Chen L Zeng S. The relationship between intolerance of uncertainty and employment anxiety of graduates during COVID-19: the moderating role of career planning. Front Psychol. (2021) 12:694785. doi: 10.3389/fpsyg.2021.694785, PMID: 34764900 PMC8576396

[82] Sun N Liu W Zheng Z. Campus outdoor environment, learning engagement, and the mental health of college students during the COVID-19 pandemic: from the perspective of students in different grades. Front Public Health. (2023) 11:1143635. doi: 10.3389/fpubh.2023.1143635 37113171 PMC10126524

[83] Xie J. Research on diagnosis and Management of Postgraduates Mental Health Status Based on BP neural network. Front Public Health. (2022) 10:897565. doi: 10.3389/fpubh.2022.897565 35619824 PMC9128816

[84] Wang CY Qi HQ Zhang JJ Zhang HC Yin ZJ. Practice and effectiveness of psychological health education and career planning and employment guidance for college students. J Heilongjiang Inst Technol. (2019) 76:70–2. doi: 10.19352/j.cnki.issn1671-4679.2019.03.015

[85] Zhang ZQ Gao CX. Research on the phenomenon of college students’ fever for civil servants in the post-epidemic era. China Univ Stud Career Guide. (2022) 14:11–7. doi: 10.20017/j.cnki.1009-0576.2022.14.002

